# Evaluation of the Change in Family Medicine Residents’ Confidence and Knowledge in Performing Basic Obstetric Ultrasound Post-training: A Prospective Study

**DOI:** 10.7759/cureus.49511

**Published:** 2023-11-27

**Authors:** Suna Soguktas, Katrina Weirauch-Engle, Nursena Kucukozyigit

**Affiliations:** 1 Family Medicine, Mohawk Valley Health System, Utica, USA; 2 Family Medicine, Sparrow Health System, Lansing, USA; 3 Family Medicine, Istanbul University School of Medicine, Istanbul, TUR

**Keywords:** education and curriculum development, maternity care, confidence, family medicine residents, training, basic obstetric ultrasound

## Abstract

Introduction

The maternity care curriculum guidelines of the American Academy of Family Physicians (AAFP) state that family medicine residents (FMRs) should demonstrate the ability to independently perform limited obstetric ultrasound (OBUS) examinations as a core skill. This study’s purpose is to examine whether basic OBUS training enhances the knowledge and confidence of FMRs in performing OBUS.

Methods

This is a Sparrow Institutional Review Board (IRB)-exempt prospective study that was completed at the Sparrow/Michigan State University (MSU) Family Medicine Residency Program (FMRP) in Michigan between December 2020 and December 2021, involving 40 residents. Assessment of knowledge and confidence in performing OBUS was completed prior to and following the training sessions.

For training, an online lecture and two separate hands-on sessions with a pregnant patient were completed. Training materials by Prof. Dr. Mark Deutchman and the University of Washington (UoW) were used. Paired T-test was used for statistical analysis, and a p-value of <0.05 was used to determine statistical significance.

Results

Thirty-two pre- and 25 post-training questionnaires were collected from the target group. Of the respondents, 92% (n=23) indicated that training increased their confidence levels in performing OBUS. The percentage of reported confidence level of 1 or 2 in performing OBUS (on a Likert scale of 5, with 5 as the highest confidence level) decreased by 60% post-training (p<0.001). Levels 3, 4, and 5 in confidence level were increased. According to the respondents, an increased confidence level in OBUS is helpful for improving trust and rapport between the provider and the patient (92%, n=23), boosting the provider’s diagnostic abilities (80%, n=20), improving patient satisfaction (76%, n=19), and decreasing healthcare costs (44%, n=11).

Conclusion

The basic OBUS training sessions improved the knowledge and confidence of residents in interpreting and performing OBUS; therefore, more OBUS training is needed during the residency.

## Introduction

Access to prenatal care plays a crucial role in enhancing birth-related outcomes. However, it is a concern that many women in rural and underserved areas across the United States face challenges in obtaining timely prenatal care, primarily because of a shortage of obstetricians [1,2].

In many rural and underserved areas in the United States, family medicine physicians provide much of the prenatal care [3]. However, most family physicians do not use prenatal ultrasound, missing a critical feature of prenatal care [4]. Ultrasound is a safe, non-invasive, and inexpensive method of care that allows immediate access to visual data [5]. According to the American Academy of Family Physicians (AAFP) Member Census (December 31, 2017), a mere 8% of AAFP physicians offer obstetric ultrasound (OBUS) imaging in their practice [6]. Such low rates may be due to limited/no OBUS training, a lack of understanding regarding technological availability, and/or a lack of ultrasound equipment [4,7].

In 2007 and 2008, the Society of Teachers of Family Medicine (STFM) Hospital Medicine and Procedural Training group suggested that all residents graduating should be capable of independently performing a basic obstetric ultrasound [8]. In addition to the recommendation from STFM, AAFP’s recommended curriculum guidelines for maternity care state that family medicine residents (FMRs) should demonstrate the ability to independently perform limited OBUS examination (i.e., fetal position, amniotic fluid index (AFI), placental location, and cardiac activity) as a core skill [9].

In medical education, training the residents for OBUS is typically conventional, involving didactics followed by supervised hands-on practice and competency assessments [10-13]. However, there is still no agreement on the exact number of scans needed to become proficient in OBUS. Some studies in primary care settings have suggested that 25-50 scans may be necessary for competency [11-14].

The potential benefits of OBUS examination performed by a family physician include the availability of immediate clinical information and assessment of urgent clinical problems, improved access and continuity of care, increased sensitivity and specificity of ultrasound examinations performed by a physician who knows the patient, and reductions in time to receive the care and healthcare cost. It may also increase the quality of maternity care/healthcare outcomes, improve the trust and rapport between the provider and the patient, and increase physician satisfaction [10].

This study’s goal was to assess the change in confidence and proficiency of the residents of the Sparrow/Michigan State University (MSU) Family Medicine Residency Program (FMRP) following OBUS training. This training style was modeled after the conventional OBUS training process [10]. Predicted changes in the professional lives and residents’ thoughts regarding the provision of ultrasound as family medicine physicians were collected as secondary data.

This article was displayed/exhibited as a poster at the Annual Mid-Michigan Regional Research Day on April 21, 2022, and had an award (third place) in the best educational research project category. Also, this article was accepted and displayed/exhibited at the AAFP-Family Centered Pregnancy Care Conference on August 17-20, 2023.

## Materials and methods

This Sparrow Institutional Review Board (IRB)-exempt study was prospectively designed to investigate data spanning from December 2020 to December 2021. This study focused on the participation of 40 residents, comprising 30 individuals from the current program and 10 from the graduating group, all of whom were affiliated with the Sparrow/MSU Family Medicine Residency Program located in Michigan.

The study was initiated with the distribution of a pre-training questionnaire, adapted from a questionnaire used in a cross-sectional study published in December 2018 in PLOS ONE, among the 40 participating residents [15]. The questionnaire served a dual purpose, comprising two sections. The first section of the questionnaire inquired about the residents’ self-assessed confidence in performing basic obstetric ultrasound. Residents were requested to express their confidence level using a numerical scale. The second section of the questionnaire contained knowledge-based questions concerning basic obstetric ultrasound. These questions were carefully selected to gauge the depth of experience and proficiency of the residents in the field of ultrasound. Residents were required to answer these questions based on their knowledge and experience.

The questionnaire was administered in an anonymous format and included an anonymous identifier field to track responses while safeguarding privacy.

A total of 32 responses were collected in response to this questionnaire. These responses constituted the baseline assessment of the residents’ confidence and knowledge levels in performing OBUS, serving as a crucial foundation for evaluating the impact of the subsequent training intervention.

The training phase of the study consisted of two distinct stages. In the initial stage, an informative session for reviewing the basic principles of OBUS was integrated into didactic education, providing residents with fundamental knowledge of OBUS. This session covered various aspects, including gestational sac (GS) measurements, crown-rump length (CRL) measurements, viability assessment, placental location, amniotic fluid index (AFI) measurement, and fetal presentation determination.

The second stage involved two hands-on training sessions, which were conducted on a standardized 20-week gestational-age patient following the obtaining of informed consent. Training materials sourced from Prof. Dr. Mark Deutchman and the University of Washington (UoW) were used to facilitate this educational phase.

To evaluate the effectiveness of the training, a post-training questionnaire adapted from the pre-training questionnaire was used. This questionnaire was designed to assess the residents’ confidence and knowledge levels in OBUS after completing the training. The primary objective of administering this questionnaire was to measure the changes in these indicators post-training. A total of 25 responses to the post-intervention questionnaire were collected for analysis.

The paired T-test was used for statistical analysis. Differences between pre- and post-training results were determined in 20 variables. Statistical significance was measured with a p-value (statistically significant p-value: <0.05).

## Results

Table 1 reveals that none of the participants indicated that the training had left their confidence entirely unaffected. Instead, 24% (n=6) stated that the training had significantly impacted their confidence, describing it as “very,” while 48% (n=12) found the training to have a “moderate” effect. A subset of seven (28%) participants reported only a “slight” influence on their confidence levels.

**Table 1:** Effect of OBUS training on the confidence of residents in performing/interpreting ultrasound OBUS: obstetric ultrasound

Answer	Responses
Very	6 (24%)
Moderately	12 (48%)
Slightly	7 (28%)
Not at all	0 (0%)

Table 2 illustrates the impact of the two training phases, namely, the informational session and the hands-on sessions, on the self-confidence levels of the participants in performing OB ultrasound. The results indicate that 92% (n=23) of the participants reported an increase in their self-confidence. Among those who attended the informational session, only one (4%) individual stated that their confidence level remained unaffected. In contrast, all participants who had participated in the hands-on training reported an increase in their confidence levels as a direct result of the hands-on session.

**Table 2:** Benefit of OBUS basic principle education/informative session and hands-on training OBUS: obstetric ultrasound

	Obstetrics ultrasound basic principles education/informative session increases my confidence level in performing/interpreting obstetrics ultrasound	Obstetrics ultrasound hands-on training increases my confidence level in performing/interpreting obstetrics ultrasound
Yes	23 (92%)	23 (92%)
No	1 (4%)	0 (0%)
Did not attend	1 (4%)	2 (8%)

In the initial survey, it was observed that 40% (n=13) of the participating residents reported having a confidence level of 1, while 46% (n=15) expressed a confidence level of 2 on a 5-point scale, where 1 indicated the lowest level of confidence and 5 the highest. None of the participants reported confidence levels of 4 or 5, with only 13% (n=4) indicating a confidence level of 3. Following the training intervention, a significant shift in participants’ confidence levels was noted. The percentage of residents with confidence level 1 decreased to 4% (n=1), and those with confidence level 2 decreased to 16% (n=4). Conversely, 56% (n=14) of the participants reported a confidence level of 3, while 24% (n=6) indicated confidence levels of 4 or 5. This shift in confidence levels was statistically significant, with a p-value of <0.001. These findings are summarized in Table 3.

**Table 3:** Confidence levels of participants in performing OBUS pre- and post-intervention 1: least, 5: most OBUS: obstetric ultrasound

	Pre-intervention	Post-intervention
Confidence level 1	13 (40%)	1 (4%)
Confidence level 2	15 (46% )	4 (16%)
Confidence level 3	4 (13%)	14 (56%)
Confidence level 4	0%	5 (20%)
Confidence level 5	0%	1 (4%)

In Table 4, a significant proportion of the participants expressed agreement regarding the various benefits associated with increased confidence in performing and interpreting OBUS. Particularly noteworthy was the enhancement in the physician-patient relationship, with 92% (n=23) of the participants concurring that heightened self-confidence in OBUS contributed to improved trust and rapport between the healthcare provider and the patient. Additionally, a substantial majority (76%, n=19) voiced their agreement that increased confidence had a positive impact on patient satisfaction, while 68% (n=17) acknowledged an improvement in physician satisfaction. Moreover, 80% (n=20) of the participants affirmed that increased self-confidence in ultrasound led to improved diagnostic abilities, and 44% (n=11) noted a decrease in patients’ healthcare costs as a result of improved self-confidence.

**Table 4:** Thoughts of participants on the benefits of increased self-confidence in performing OBUS in various contexts OBUS: obstetric ultrasound

Increased confidence in performing/interpreting obstetrics ultrasound is helpful for…	Participants in agreement
…improving the satisfaction of the patient	19 (76%)
…improving the trust and rapport between the provider and the patient	23 (92%)
…improving the satisfaction of the provider	17 (68%)
…boosting my diagnostic abilities	20 (80%)
…decreasing healthcare costs	11 (44%)

The accuracy rates in answering knowledge questions within the questionnaire exhibited significant changes as illustrated in Table 5. Prior to the training, 47% (n=15) of the participants answered questions correctly, while post-training, this figure increased to 68% (n=17). Conversely, the percentage of incorrect answers decreased from 34% (n=11) pre-training to 20% (n=5) post-training. Moreover, the proportion of unknown answers decreased from 19% (n=6) prior to the training to 12% (n=3) after the training. The change in the level of knowledge regarding OBUS was statistically significant, with the highest p-value among the knowledge questions being 0.014.

**Table 5:** Pre- and post-intervention knowledge scores

	Responses pre-intervention	Responses post-intervention
Correct	15 (47%)	17 (68%)
Incorrect	11 (34%)	5 (20%)
Unknown	6 (19%)	3 (12%)

Furthermore, the study demonstrated a shift in participants’ expectations regarding the utilization of obstetric ultrasound (OBUS). Pre-training, 34.3% (n=11) of the participants expressed an intention to use OBUS in their practice and play a minor or moderate role in providing OBUS. Post-training, this percentage increased substantially to 64% (n=16). Although the impact on participants expecting to play a major role was relatively modest, it only decreased by approximately 3% after the training with the p-value being 0.8.

## Discussion

Obstetric ultrasound has emerged as a fundamental tool in prenatal care, offering valuable insights into the well-being of both the mother and the developing fetus. It is a safe, non-invasive, and inexpensive method of care that allows immediate access to visual data [5]. In rural and underserved areas, the provision of prenatal care is predominantly led by family medicine physicians. Various institutions have made recommendations regarding the training methods to enhance maternity care in these areas [3]. The most widely advocated approach in medical education has been the conventional model, which typically involves didactic sessions followed by hands-on training and competency assessments [10-13]. Both the Society of Teachers of Family Medicine (STFM) and the American Academy of Family Physicians (AAFP) emphasize that, upon graduation, all family medicine residents should possess the ability to independently perform basic obstetrical ultrasound (OBUS) [8,9].

While there is currently no consensus on the exact number of scans required to attain competency in OBUS, several studies conducted in family medicine education settings have suggested that a range of 25-50 scans may be necessary to achieve this competency [11-14].

There are numerous advantages associated with the acquisition of competence in OBUS for family medicine providers, which include but are not limited to the following: the availability of immediate clinical information for assessing urgent clinical issues (i.e., the viability of the fetus, amniotic fluid volume, placental location, and fetal presentation in labor process), enhanced access to and continuity of care, increased satisfaction among both patients and physicians, and a reduction in healthcare costs [10].

In this research, our primary objective was to assess whether increased training has an impact on the confidence levels of family medicine providers when delivering maternity care and if it enhances their proficiency in performing and interpreting basic obstetric ultrasound. The results of our study indicate that both confidence levels and knowledge have been positively affected as a result of this increased training, which increases the satisfaction of the providers and the patients and decreases healthcare costs. This improvement in skills and confidence is expected to have a beneficial impact on the quality of maternity care provided by family medicine providers, particularly in underserved and rural areas across the United States.

The small sample size and decreased number of participants post-survey, limited access to patients for hands-on sessions, and limited time for teaching and for residents to practice ultrasound were some limitations of this study. Future study opportunities include evaluating the effectiveness of different OBUS teaching techniques for FMRs, analyzing potential correlations between confidence in performing OBUS and patient satisfaction, and assessing the compatibility of different ultrasound technologies/devices with family medicine practitioners.

## Conclusions

This study indicates that the confidence in performing OBUS and the knowledge of OBUS of participating residents increases dramatically post-training. Additional positive effects were reported by the participants, including a better relationship with their patients and increased satisfaction regarding their jobs. Furthermore, and perhaps most importantly, a higher percentage of participants expected to provide OBUS care following their graduation from residency, which is invaluable for improving prenatal care in especially underserved and rural areas. Overall, the results of the study suggest that OBUS training in the family medicine residency setting results in greater confidence, knowledge, and anticipated utilization of OBUS in clinical practice and therefore may be an important tool to expand prenatal care in low-access settings.
